# Impact of grade on workup of rectal neuroendocrine tumors: a retrospective cohort study: Grade impact on workup of rectal NETs

**DOI:** 10.1186/s12957-024-03379-5

**Published:** 2024-04-16

**Authors:** Akie Watanabe, Sabrina Rai, Lily Yip, Carl J. Brown, Jonathan M. Loree, Heather C. Stuart

**Affiliations:** 1 Gordon & Leslie Diamond Health Care Centre, Vancouver General Hospital, University of British Columbia, 2775 Laurel St., 5th Floor, 899 W 12th Ave, Vancouver, BC V5Z 1M9 Canada; 2 St. Paul’s Hospital, University of British Columbia, 1081 Burrard Street, Vancouver, BC V6Z 1Y6 Canada; 3 BC Cancer Agency, 600 W 10th Ave, Vancouver, BC V5Z 4E6 Canada

**Keywords:** Rectal neuroendocrine tumors, Preoperative staging, Chromogranin A, Urine 5-HIAA, Operative decision-making, Tumor grade, Recurrence, Survival

## Abstract

**Background:**

Rectal neuroendocrine tumors (RNETs) are often discovered on screening colonoscopy. Indications for staging and definitive resection are inconsistent in current guidelines. We evaluated the role of grade in guiding staging and procedural decision-making.

**Methods:**

Patients with biopsy confirmed RNETs between 2004 and 2015 were reviewed. Baseline characteristics, staging investigations (biochemical and imaging), and endoscopic/surgical treatment were recorded. Associations between grade, preoperative staging, interventions, and survival were determined using Fisher-Freeman-Halton Exact, log-rank, and Kaplan-Meier analysis.

**Results:**

Amongst 139 patients with RNETs, 9% were aged ≥ 75 years and 44% female. Tumor grade was: 73% grade 1 (G1), 18%, grade 2 (G2) and 9% grade 3 (G3). Staging investigations were performed in 52% of patients. All serum chromogranin A and 97% of 24-hour urine 5-hydroxyindoleacetic acid tests were normal. The large majority of staging computed tomography (CT) scans were negative (76%) with subgroup analysis showing no G1 patients with CT identified distant disease compared with 38% of G2 and 50% of G3 patients (*p* < 0.001). G1 patients were more likely to achieve R0/R1 resections compared to G2 (95% vs. 50%, *p* < 0.001) and G1 patients had significantly better 5-year overall survival (G1: 98%, G2: 67%, G3: 10%, *p* < 0.001).

**Conclusion:**

Tumor grade is important in preoperative workup and surgical decision-making. Biochemical staging may be omitted but staging CT should be considered for patients with grade ≥ 2 lesions. Anatomic resections should be considered for patients with grade 2 disease.

**Supplementary Information:**

The online version contains supplementary material available at 10.1186/s12957-024-03379-5.

## Background

Rectal neuroendocrine tumors (RNETs) are most commonly detected on screening colonoscopy with studies reporting a 0.05–0.07% prevalence [1]. Detection is often incidental, and an R0 resection is seldom achieved during initial endoscopic biopsy [2]. However, the prognosis is still favourable and 5-year overall survival (OS) is reported between 86–91% [3, 4]. The role of staging and definitive management for RNETs found on biopsy are not well defined and current guidelines are inconsistent [1, 5, 6]. .

While preoperative staging can heavily impact management strategies for other malignancies (e.g. gastric [7], colorectal adenocarcinomas [8]), incidentally discovered RNETs rarely metastasize [3] so the utility of staging is unclear. Biochemical assessment following diagnosis of a gastroenteropancreatic (GEP) NET often includes 24 hour urine 5-hydroxyindoleacetic acid (5-HIAA) [9] and serum chromogranin A (CgA) [10, 11], which can be elevated in tumors of certain primary sites. Imaging with computed tomography (CT), magnetic resonance imaging (MRI), somatostatin receptor scintigraphy (SRS) [5, 6] or DOTATOC positron emission tomography (PET)/CT can have a role in assessing GEP-NETs but their appropriateness in staging RNETs is not well established. Indications for staging are discordant with the North American Neuroendocrine Society (NANETS) recommending against staging < 20 mm lesions [5] and the European Neuroendocrine Society (ENETS) recommending staging > 10 mm lesions [6].

Definitive management can include endoscopic mucosal resections (EMR), endoscopic submucosal dissection (ESD), transanal endoscopic surgery (TES), open transanal surgical resections, or anatomic resections (low anterior resection, proctectomy, etc.) [2, 5, 6, 12]. Both NANETS and ENETS recommend endoscopic resection for tumors < 10 mm and anatomic resections > 20 mm [5, 6], but the optimal intervention for tumors between 10 and 20 mm is unclear [1]. A systematic review from 2014 suggests local excisions to be safe in lesions up to 16 mm [13] while another population-based study suggests its safety only in T1 tumors < 15 mm [14].

Lack of consensus and understanding on the optimal management of RNETs has led to variable practices. While guidelines for other malignancies such as breast cancer incorporate grade into clinical prognostic staging [15], the American Joint Committee on Cancer (AJCC) TNM staging as well as current guidelines for RNETs emphasize tumor size alone and do not consider grade despite it is a well-known prognostic factor [4, 16–18]. In this study, we explore associations between grade, preoperative staging investigations, and procedural interventions to improve guideline consistency.

## Methods

### Study population

Data were collected retrospectively on patients diagnosed with RNETs from 2002 to 2015 and obtained from two sources. The first is a pathology database at a hospital in the largest health authority in British Columbia with a catchment of 1.25 million people where all pathology cases diagnosing RNETs were included. The second source was from the British Columbia Cancer Agency Information System (CAIS) that included all RNET cases referred to one of 6 regional cancer centres in British Columbia.

### Study outcomes and variables

Primary outcomes included the frequency and modality of staging investigations and procedural interventions performed. Secondary outcomes included cumulative incidence of recurrence and OS. Baseline patient characteristics included age (dichotomized as ≥ 65 years or less) [19] and sex. Race and ethnicity are not documented in patient files; therefore, we were unable to retrospectively collect this information. Tumor characteristics included pTNM stage, tumor size (mm), and lymphovascular invasion (LVI). Grade was reported from the final postoperative pathology when applicable, and otherwise from preoperative biopsies. All tumors with Ki67 index > 20% or otherwise indicated on the pathology report were categorized as grade 3 RNETs. Distinction between well differentiated grade 3 NETs and grade 3 neuroendocrine carcinomas (NECs) was not established during the study period and thus not recorded [20].

Frequency and positivity of preoperative staging investigations classified as biochemistry (24-hour urine 5-HIAA and serum CgA) and/or imaging (CT, MRI, functional SRS, metaiodobenzylguanidine (MIBG) scan, and fluorodeoxyglucose positron emission tomography (^18^F-FDG PET/CT)) performed after the date of diagnosis and before definitive procedure or surveillance follow-up were collected. Location of disease identified on imaging was recorded and classified as local, regional or distant disease.

Procedural intervention was defined as endoscopy and/or surgical intervention. Receipt of endoscopy included colonoscopy or flexible sigmoidoscopy. Local resections included TES or open transanal excisions under anesthesia. Anatomic resections included low anterior resection, abdominoperineal resection (APR), proctectomy, or transanal total mesorectal excision (taTME). Reasons for performing an intervention were collected based on surgeon description or assumed from margin status reported on initial pathology if a description was unavailable. For those who had a procedure, completeness of resection (R0/R1, R2) was recorded. R0 and R1 resections were placed in the same category as previous research suggests that patients with low grade RNETs resected with positive microscopic margins had similar prognosis to those with negative margins [21].

### Statistical analysis

Baseline and tumor characteristics were analyzed using descriptive statistics and associations with grade were determined using Fisher-Freeman-Halton Exact analyses. Missing data censored the patient profile in subsequent analyses and data imputation was not performed.

Frequency and positivity of staging investigations and types of interventions performed were reported using descriptive statistics. Associations between preoperative staging investigations, procedural interventions and grade were determined using Chi-square or Fisher’s exact analyses. Statistical significance was defined as *p* < 0.05.

Time to cumulative incidence of recurrence, including local, regional and/or distant recurrence, was calculated from the date of last procedure to the date of recurrence or last contact in patients who had an R0/R1 resection. OS was defined as the date of diagnosis to the date of death from any cause or last contact. Cumulative incidence of recurrence and OS between patients with grade 1, 2, or 3 disease was estimated using Kaplan-Meier analysis. Statistical significance was established using a two-sided log-rank test and defined as *p* < 0.05. Statistical analyses were completed with SPSS v 28.0.

## Results

Among 139 patients diagnosed with RNETs, 32% (44/139) were aged ≥ 65 years and 44% (61/139) were female. Grade 1, 2, and 3 disease were present in 73% (83/113), 18% (20/113), and 9% (10/113) of patients, respectively (Table 1). There were 1% of grade 1 patients that had metastatic disease at diagnosis (1/83) compared to 45% (9/20) and 80% (8/10) in grade 2 and 3 patients, respectively (*p* < 0.001) (Table 1).

**Table 1:** Baseline characteristics

Covariates	Overall (%)	Grade 1 (%)	Grade 2 (%)	Grade 3 (%)	*P*
	*N* = 139	*N* = 83	*N* = 20	*N* = 10	
**Age**					0.06
≥ 65	44 (32)	23 (28)	11 (55)	2 (20)	
**Sex**					0.35
Female	61 (44)	41 (49)	7 (35)	3 (30)	
**pT Stage** ^**a**^					0.003
X	18 (15)	9 (12)	0 (0)	2 (50)	
pT1	72 (62)	52 (68)	9 (60)	1 (25)	
pT2	18 (15)	13 (17)	1 (7)	1 (25)	
pT3	9 (8)	3 (4)	5 (33)	0 (0)	
pT4	0 (0)	0 (0)	0 (0)	0 (0)	
**pN Stage** ^**a**^					0.002
X	96 (85)	66 (90)	7 (50)	4 (100)	
pN0	10 (9)	6 (8)	2 (14)	0 (0)	
pN1	5 (4)	1 (1)	4 (29)	0 (0)	
pN2	2 (2)	0 (0)	1 (7)	0 (0)	
**Metastatic disease at diagnosis**					< 0.001
Yes	22 (16)	1 (1)	9 (45)	8 (80)	
**Diameter** ^**a**^					0.23
< 10 mm	81 (90)	57 (92)	10 (77)	1 (100)	
10–20 mm	8 (9)	5 (8)	3 (23)	0 (0)	
≥ 20 mm	1 (1)	1 (0)	0 (0)	0 (0)	
**LVI** ^**a**^					0.04
Positive	7 (11)	3 (6)	3 (27)	1 (50)	
**Status**					< 0.001
Dead	27 (19)	5 (6)	10 (50)	9 (90)	
**Recurrence**	*N* = 110	*N* = 77	*N* = 10	*N* = 1	0.01
Yes	12 (11)	5 (6)	4 (40)	0 (0)	

^**a**^Missing data present; *p* < 0.05; *LVI* Lymphovascular invasion

### Preoperative staging

**Fig. 1:**
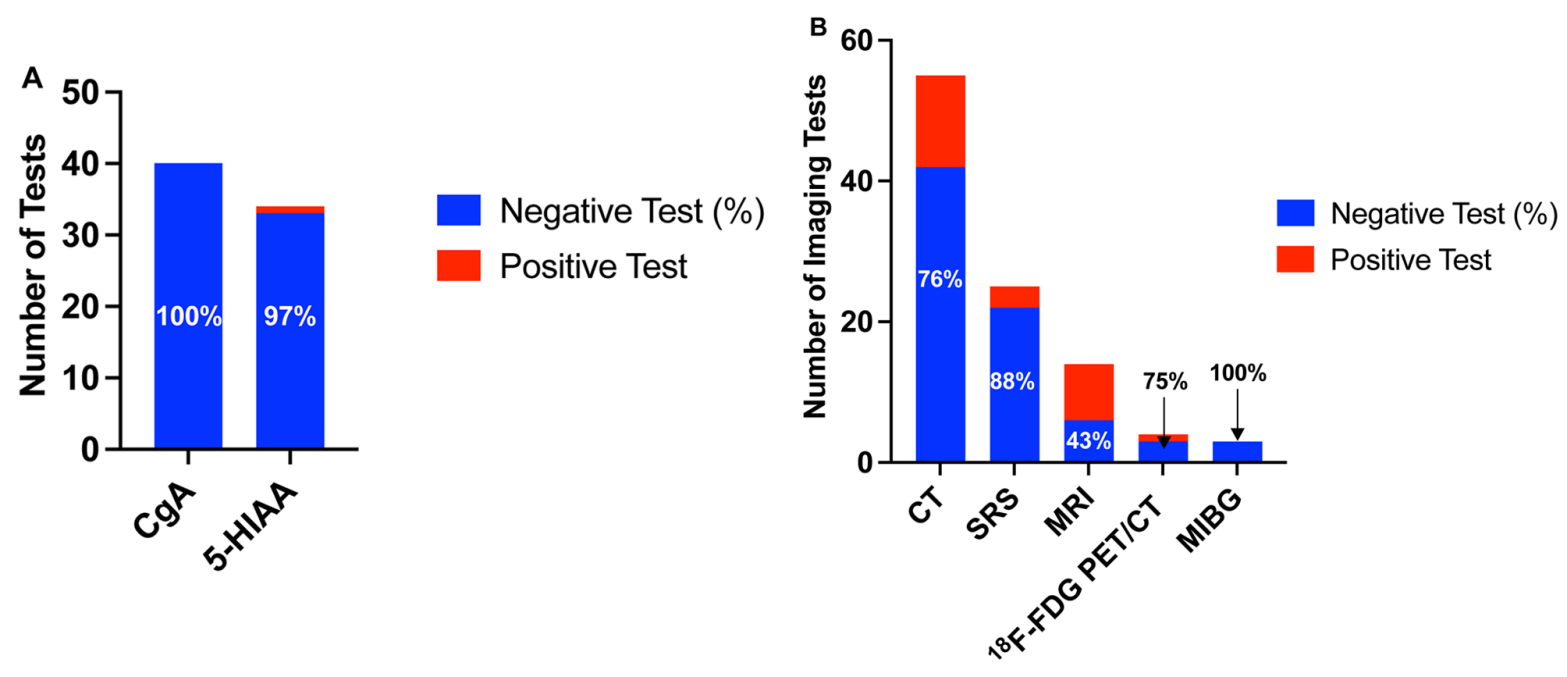
Population that underwent preoperative staging. (**A**) Number of patients who underwent biochemical staging out of 124. (**B**) Number of patients who underwent staging imaging out of 124. *The total number of patients who were eligible for preoperative biochemistry and imaging was 124

Of 124 patients that were diagnosed with an RNET on colonoscopy, 52% (64/124) had staging investigations. Amongst patients with reported diameters, 9% (8/90) and 1% (1/90) had a lesion 10–20 mm and ≥ 20 mm, respectively. Serum CgA was performed in 32% (40/124, 100% negative, 40/40) and urine 5-HIAA was performed in 27% (34/124, 97% negative, 33/34) of patients (Fig. 1a). One patient with a 2 mm, grade 1 tumor had a positive urine 5-HIAA (54 umol/d, ref < 50 umol/d) but not found to have metastatic disease at diagnosis. Grade data were available in 100 patients where higher grade disease was associated with provider-arranged urine 5-HIAA investigations (*p* = 0.007) but not with serum CgA (*p* = 0.09) (Table 2).

**Table 2:** Investigations ordered as staging for patients with R-NETs based on grade

Investigations	Grade 1 (%)^a^	Grade 2 (%)^a^	Grade 3 (%)^a^	*P*
**Biochemistry**	***N*** ** = 81**	***N*** ** = 15**	***N*** ** = 4**	
Serum CgA	26 (32)	9 (60)	1 (25)	0.09
Urine 5-HIAA	20 (25)	10 (67)	1 (25)	0.007
**Imaging**	***N*** ** = 81**	***N*** ** = 15**	***N*** ** = 4**	
CT	35 (43)	8 (53)	2 (50)	0.77
MRI	6 (7)	5 (33)	3 (75)	< 0.001
SRS	19 (24)	3 (20)	1(25)	1.00
MIBG	3 (4)	0 (0)	0 (0)	1.00
^18^F-FDG PET/CT	2 (3)	1 (7)	1 (25)	0.08

^**a**^Missing data present; *CgA* chromogranin A; *5-HIAA* 5-hydroxyindoleacetic acid; *CT* computed tomography; *MRI* magnetic resonance imaging; *SRS* somatostatin receptor scintigraphy; *MIBG* metaiodobenzylguanidine; ^*18*^*F-FDG PET/CT* fluorodeoxyglucose positron emission tomography; *p* < 0.05

**Fig. 2:**
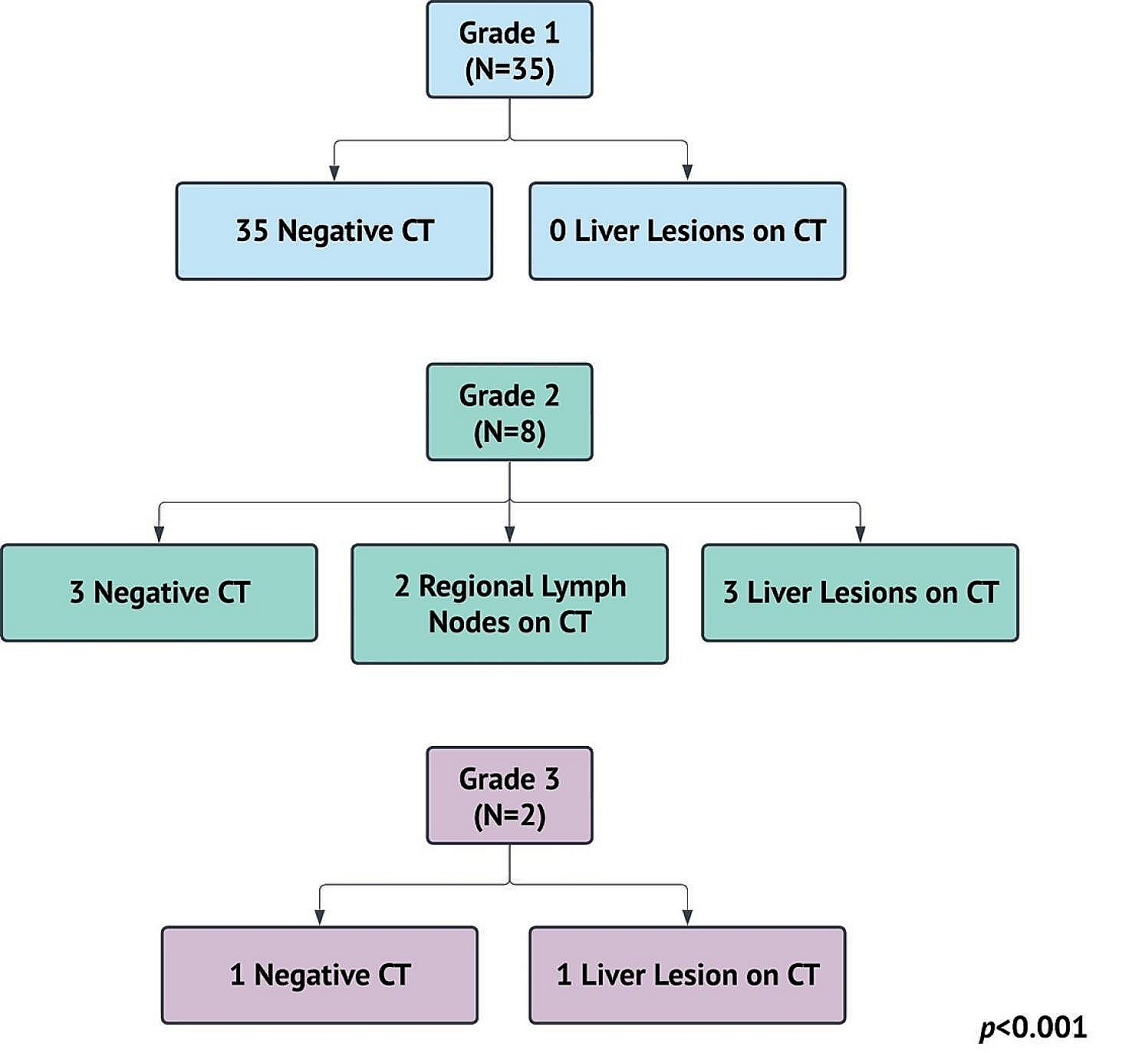
Metastasis on CT scan stratified by grade *CT* computed tomography

CT was the most frequently ordered staging investigation at 44% (55/124) with 76% (42/55) showing negative results. Amongst the 13 CTs with positive findings, 54% (7/13) showed liver metastasis, 38% (5/13) local disease, and 15% (2/13) regional disease (Additional file 1). There was no confirmed pulmonary metastasis on chest CT. SRS was the second most ordered imaging modality at 20% (25/124), followed by MRI at 11% (14/124) with negative results in 88% (22/25) and 43% (6/14) of scans, respectively (Fig. 1b). Amongst MRIs performed, 93% (13/14) were pelvic MRIs (Fig. 1b) of which 62% (8/13) were positive detecting local or regional disease (Additional file 1). All staging CTs were negative in patients with grade 1 disease, while 38% (3/8) detected liver metastasis for grade 2, and 50% (1/2) for grade 3 disease (*p* < 0.001) (Fig. 2). Provider arrangement of imaging was not associated with grade except for MRI (*p* < 0.001) which was more likely to be ordered with higher grade disease (Table 2).

### Procedural and surgical intervention

**Fig. 3:**
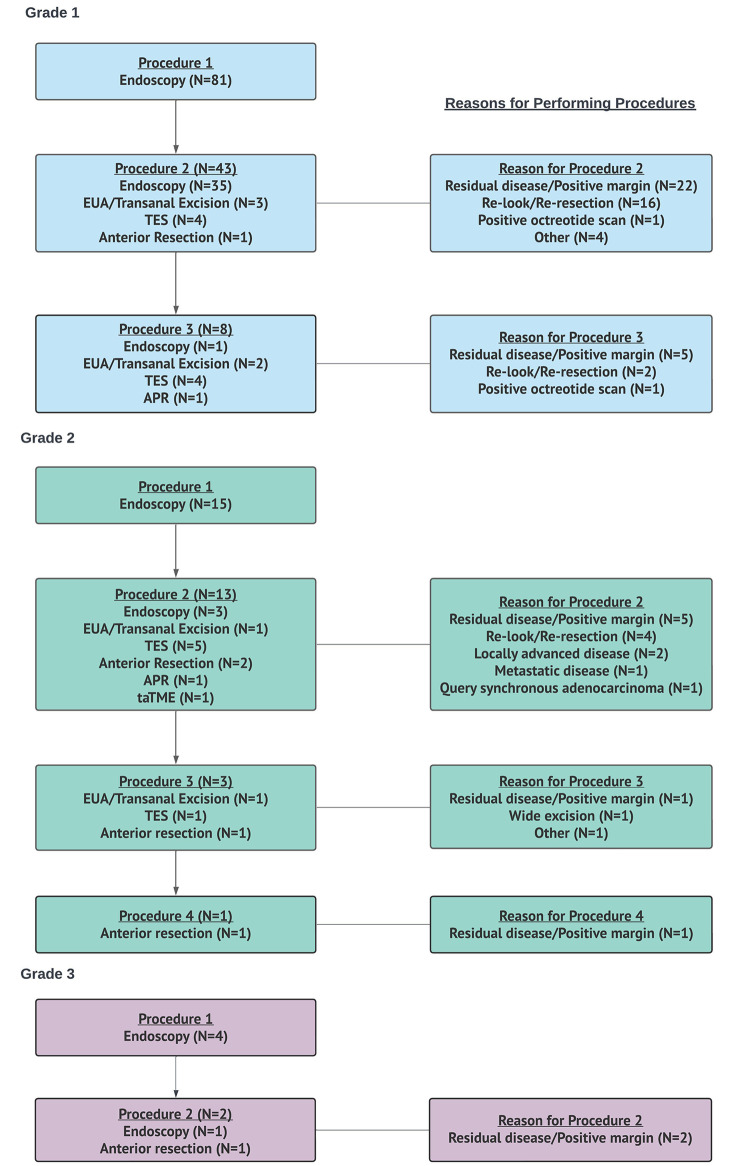
Procedural flow by tumour grade *EUA* Examination under anesthesia; *TES* Transanal endoscopic surgery; *APR* abdominoperineal resection; *taTME* transanal total mesorectal excision

Overall, the rate of local resection increased with grade; 16% (13/83) grade 1 and 40% (8/20) grade 2 (*p* = 0.01). Grade 1 patients underwent fewer anatomic resections compared to grade 2 patients (2% (2/83) vs. 30% (6/20), *p* < 0.001). R0/R1 resection margins were achieved with procedural interventions in 98% (77/79), 67% (10/15), and 25% (1/4) of grade 1,2, and 3 patients, respectively (*p* < 0.001) (Table 3). Amongst 124 patients who underwent initial colonoscopy, 100 patients had reported grade data. Initial colonoscopy alone was performed in 42 patients, while 58 patients had a 2nd, 11 had a 3rd, and 1 had a 4th procedure (Fig. 3). The most common reasons for performing recurrent procedures were residual disease or positive margins from prior procedure at 51% (35/68) followed by a re-look to see if all the disease was removed at 32% (22/68) (Fig. 3).

**Table 3:** Association between grade and intervention

Resection	Grade 1 (%) *N* = 83	Grade 2 (%) *N* = 20	Grade 3 (%) *N* = 10	*P*
**Any Procedure**				< 0.001
Yes	81 (98)	15 (75)	4 (40)	
^**a**^ **Local Resection**				0.01
Yes	13 (16)	8 (40)	0 (0)	
^**b**^ **Anatomic/Surgical Resection**				< 0.001
Yes	2 (2)	6 (30)	1 (10)	
**Resection Completeness**				< 0.001
R0/R1	77 (98)	10 (67)	1 (25)	
R2	2 (2)	5 (33)	3 (75)	

^a^Local resection includes examination under anesthesia, transanal excision, transanal endoscopic surgery

^b^Anatomic/surgical resection includes anterior resection, abdominoperineal resection, transanal total mesorectal excision

*p* < 0.05

### Recurrence and survival

**Fig. 4:**
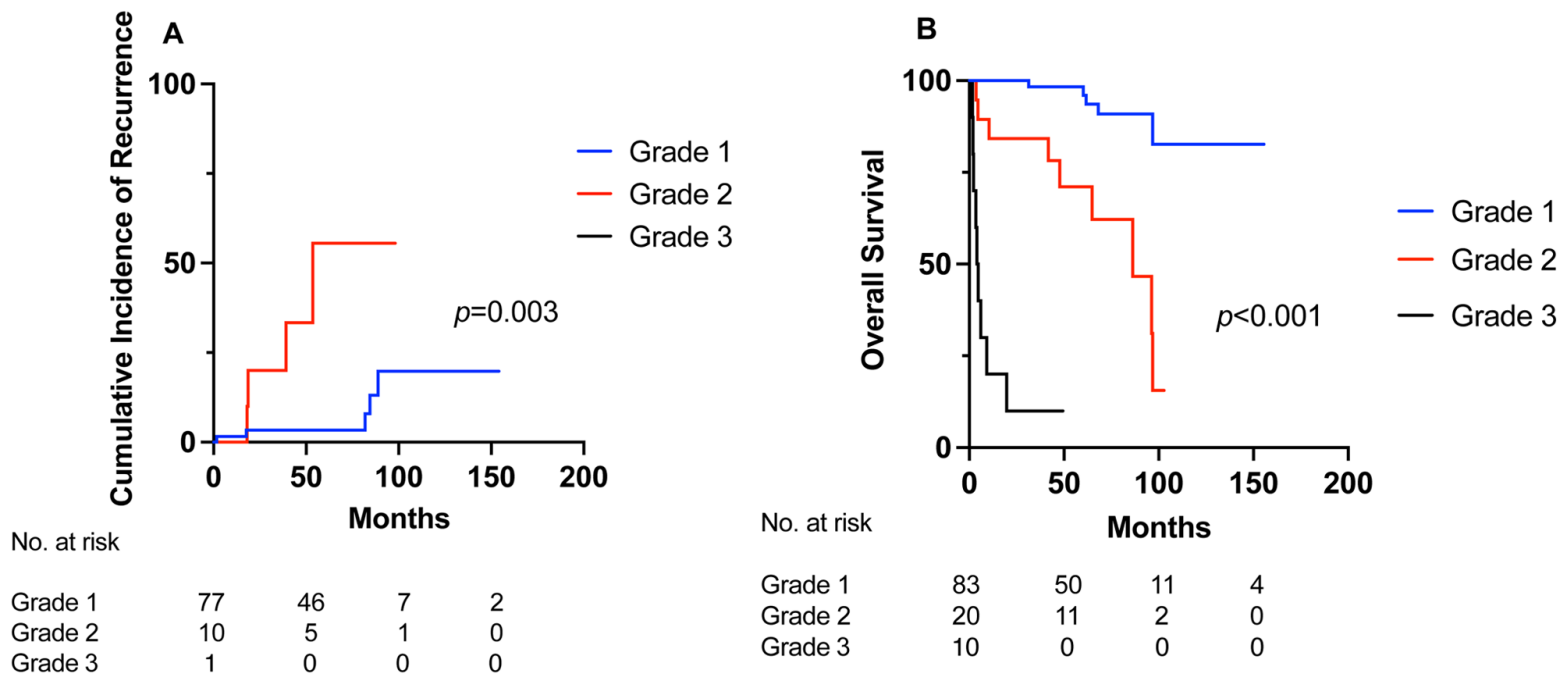
Impact of grade on recurrence and overall survival. (**A**) Cumulative incidence of recurrence in rectal NETs with R0/R1 resection. (**B**) Overall survival

In patients who underwent an R0/R1 resection, 9% (13/139) recurred. The estimated 5-year cumulative incidence of recurrence was 3% and 56% for grade 1 and 2 disease, respectively (*p* = 0.003) (Fig. 4a). Including all patients, the median OS was unmeasurable for grade 1 disease, but was 86 months for grade 2, and 4 months for grade 3 disease (*p* < 0.001). The 5-year OS was significantly longer in grade 1 disease, followed by grade 2, and grade 3 disease (98% vs. 67% vs. 10%, *p* < 0.001) (Fig. 4b).

## Discussion

In this study, the impact of grade on staging investigations and treatment of RNETs was explored. Our results suggest that there is minimal role in biochemical staging even though it is ordered excessively by providers irrespective of grade. Staging imaging may be useful in grade ≥ 2 lesions, as these patients had more metastasis at diagnosis, higher recurrence risk, and worse OS. Patients with grade 2 lesions also underwent more anatomic resections, highlighting grade as a potential factor in guiding treatment decision-making. Overall, grade is a valuable marker that should be consistently incorporated into early RNET staging and management guidelines.

RNETs are often small with low risk features and distant metastases are rare [1] with some studies quoting a rate as low as 4.2% at the time of diagnosis [3]. Our study reported a higher rate of 19%, which is likely influenced by a larger population of grade ≥ 2 disease. When stratified by grade, 1% and 45% of patients with grade 1 and 2 disease, respectively, had distant metastasis at diagnosis which parallels the increasing trend of 2.1% and 31.4% (*p* < 0.001) reported in literature [22]. The more aggressive tumor biology seen in grade ≥ 2 lesions [22] suggests a need for complete staging and definitive management strategies.

There is variability and lack of clarity in current guidelines for timing, modality and indication of staging for RNETs [5, 6]. NANETS recommend no staging for lesions < 20 mm while ENETS and the National Comprehensive Cancer Network (NCCN) recommend no staging for lesions < 10 mm [5, 6, 23]. In our study, 52% underwent some form of staging even though only 10% of patients had lesions ≥ 10 mm. Apart from urine 5-HIAA and MRI, staging investigations were not associated with grade suggesting a lack of provider understanding of RNET biology. Better adherence to guidelines and incorporation of grade into staging strategies could help stratify resources to improve diagnostic efficacy.

Twenty-four-hour urine 5-HIAA and serum CgA are often performed in conjunction with a diagnosis of a NET. Their role in staging RNETs is controversial and there is limited evidence to support their use. In our study, 27% and 32% of patients underwent urine 5-HIAA and serum CgA staging, respectively, but 97% and 100% of results were negative. Hindgut NETs rarely produce serotonin [24]; therefore, an elevated 24 h urine 5-HIAA test is often non-specific [25] and has no known prognostic value [9]. Serum CgA may have a limited role in predicting extent of disease in small bowel NETs [11]; but its use in diagnosis and prediction of recurrence in RNETs is unclear [10]. False positive results are common, making elevated values, especially those only mildly above the upper limit of normal, difficult to interpret [5, 25]. Our study shows no utility for biochemical staging with urine 5-HIAA or serum CgA for RNETs, regardless, which is concordant with current literature [5, 6, 10, 24, 25].

Imaging plays an important role in staging disease and is necessary to guide subsequent management in many malignancies. CT is the most common imaging modality performed for staging in our study with a 24% rate of disease detection. Liver metastases were identified in 54% of positive CT scans with the majority (57%) in patients with grade 2 or 3 disease while 43% of positive scans did not have grade documented. Of patients with grade 1 disease confirmed from endoscopy who also underwent CT staging, none were found to have liver metastases. Only one patient with grade 1 disease had peritoneal metastasis at diagnosis, but this was identified during surgery and not on the CT scan; therefore, staging CT in this patient would not have been useful. CT is recommended as an initial staging modality by ENETS [6] and other studies [18] because of its accessibility, low cost, and reasonable sensitivity for detection of liver metastases (43–80%) [26, 27]. Based on our findings, we recommend that all patients with grade 2 or 3 RNETs undergo staging with CT of the abdomen and pelvis. Lung metastases in GEP-NETs are uncommon [28, 29]; therefore, staging CT chests are not recommended.

Other imaging modalities for staging were also explored in our study. Amongst 13 patients who received a pelvic MRI, local or regional disease was detected in 62%. As lesions < 10 mm and > 10 mm have a 25% [30] and 61% [31] incidence of regional nodal metastases, respectively, pelvic MRIs can be a useful method in detecting regional disease. As regional lymph node metastases are a poor prognostic factor in RNETs [30–33], early detection is crucial to guide subsequent management. While 93% of MRIs were limited to the pelvis in our study, and the only abdominal MRI did not identify any metastases, MRIs are thought to have a greater sensitivity in detecting metastatic liver disease (71–99% [27]) compared to CT. ENETS guidelines list MRI as a potential first line modality for tumors > 10 mm [6, 18], suggesting its role in staging high grade lesions. SRS is another imaging modality commonly used for staging NETs, and while it was frequently performed in our study (18%), positivity rates were low (3%). Somatostatin receptors are more commonly found in low grade compared to high grade GEP-NETs and NECs [34], which can be useful for guiding functional imaging. NANETS guidelines emphasize its low sensitivity [5] relative to other imaging and does not recommend it as a staging modality. Similarly, MIBG scans may have utility in imaging pheochromocytomas or paragangliomas but have limited role in RNET imaging [28]. While both ENETS and NANETS highlight endoscopic ultrasound (EUS) evaluation of RNETs [5, 6], its role was minimally explored in our study as only 5 patients underwent EUS after initial endoscopy. The majority of studies suggest high (close to 100%) accuracy in EUS assessment of depth of invasion; but it may be limited in its ability to detect residual disease following biopsy [35]. EUS may have a role in surveillance for recurrence [36].

An important consideration in the management of RNETs is the role of further excision (local or anatomical) following initial diagnosis. The presence and/or location of microscopic residual disease can be difficult to discern after initial polypectomy making further intervention difficult. In our study, the presence of positive margins on initial biopsy was the most common reason for further intervention (51%) with 54% identifying residual malignant disease. However, a study by Sun et al. (2023) showed that the presence of positive margins after ESD for RNETs did not impact 5-year progression free survival or OS [37]. In our study, amongst 33% who underwent intervention after biopsy without clear documentation of positive margins, 50% detected malignant disease on final pathology. Further procedural decision-making should not solely rely on margin status but also take into consideration other tumor characteristics.

Current ENETS, NANETS and NCCN guidelines recommend local or endoscopic resections for tumors ≤ 20 mm and anatomic resections for tumors > 20 mm with a strong emphasis on tumor size [5, 23, 38]. Although ENETS and NCCN factors grade into decision-making when secondary resection is incomplete, it is not emphasized in initial procedural planning [6, 23]. In our study, the rate of local and anatomic resections was lower in patients with grade 1 compared to grade 2 disease. Within patients with grade 1 disease, local surgical resections were more commonly performed than anatomic resections. Several studies have shown that endoscopic resection techniques are adequate in completely resecting localized RNETs with one study quoting a 99% en bloc section rate [12]. A large Canadian study showed that TEM is effective for primary excisions and completion re-excisions with low recurrence rates (5%) [39]. Endoscopic full thickness resections (eFTR) are comparable to TEM [40], and are more effective than repeat biopsy of the scar [35] in achieving progression free survival and OS [37]. Endoscopic resection may be more efficient and less invasive compared to transanal resections [40] and could be considered for RNETs with low grade disease. In comparison, patients with grade 2 disease had a higher rate of R2 resections compared to those with grade 1 disease (33% vs. 2%) suggesting a need for radical interventions [30, 32]. They also underwent more anatomic than local resections, despite all lesions were smaller than 10 mm, suggesting that surgical decision-making should incorporate grade in addition to tumor size. Lastly, declining rates of 5-year OS based on grade (grade 1: 98%, grade 2: 67%, grade 3: 10%) parallels that reported by Weinstok et al. (grade 1: 87.7%, grade 2: 47.6%, grade 3: 33.3%) [41], which further highlights its role in the preoperative treatment algorithm [4, 41].

While tumor size is known to be a significant prognostic factor [5, 6, 42], recent studies have suggested that it may be insufficient in guiding staging, surgical decision-making [43] and prognosis [44]. RNETs ≥ 20 mm are scarce, making it difficult to assess statistical outcomes for this population [43]. Additionally, evaluation of tumor size at diagnosis may not be accurate if only a biopsy or partial polypectomy was performed. Few studies in other NETs such as appendiceal and gastroduodenal [45, 46] are exploring the incorporation of grade into their management algorithms. Other cancers such as breast have already incorporated grade into clinical prognostic staging [15]. With increasing literature evidence that grade is an important prognostic indicator for RNETs [22, 47], consideration of its addition to management algorithms is likely warranted.

### Limitations

This study is limited by its retrospective design. Despite collecting 13 years’ worth of data, the population size was limited due to the low incidence of RNETs; therefore, it was not possible to perform regression or prediction analysis. A portion of our patient data were collected from regional cancer centres, where more aggressive disease tends to be referred, which may explain our higher rates of metastasis. We were unable to collect data on all patients in the province due to limitations in data sharing. Data for grade, tumor size, depth of invasion and specimen margin were often missing from old pathology reports, which limited the population size available for analysis. Results must also be interpreted with the limitation that data collection was performed prior to the 2019 World Health Organization change in grading criteria [48]. Six patients with grade 3 tumors did not undergo any intervention limiting their assessment. EMR or ESD data is not included as these were not commonly performed during the study period. Indication for further procedures may be inaccurate as margin data were often missing from pathology reports and documentation by physicians were often vague.

## Conclusion

The spectrum of disease within RNETs is poorly characterized and improved understanding of this should guide assessment and management. Based on our data, we recommend consideration of omitting biochemical staging for RNETs. Future guidelines may consider incorporating grade, in addition to tumor size, as a useful factor in determining staging and surgical approach for RNETs. Preoperative imaging with CT and/or MRI for local and distant disease should be considered for patients with grade ≥ 2 lesions. Anatomic resections should be considered for patients with grade 2 disease while those with grade 1 disease may benefit from advanced endoscopic resection. Future prospective investigations are required to confirm our findings before possible incorporation into guidelines.

### Electronic supplementary material

Below is the link to the electronic supplementary material.

**Supplementary Material 1: Supplementary Table 1.** Location of disease in staging imaging with positive findings. Description: where different types of disease spread was detected by different imaging modalities

## Data Availability

No datasets were generated or analysed during the current study.

## Abbreviations

5-HIAA: 5-hydroxyindoleacetic acid
^18^F-FDG PET/CT: Fluorodeoxyglucose positron emission tomography
AJCC: American Joint Committee on Cancer
APR: Abdominoperineal resection
CgA: Chromogranin A
CT: Computed tomography
eFTR: Endoscopic full thickness resection
EMR: Endoscopic mucosal resection
ENETS: European Neuroendocrine Society
ESD: Endoscopic submucosal dissection
EUS: Endoscopic ultrasound
GEP: Gastroenteropancreatic
LVI: Lymphovascular invasion
MIBG: Metaiodobenzylguanidine
MRI: Magnetic resonance imaging
NANETS: North American Neuroendocrine Society
NCCN: National Comprehensive Cancer Network
NEC: Neuroendocrine carcinoma
OS: Overall survival
PET: Positron emission tomography
RNET: Rectal neuroendocrine tumor
SRS: Somatostatin receptor scintigraphy
taTME: Transanal total mesorectal excision
TES: Transanal endoscopic surgery
