# Supplementary material for: Impact of grade on workup of rectal neuroendocrine tumors: a retrospective cohort study: Grade impact on workup of rectal NETs
Source: World J Surg Oncol. 2024 Apr 16;22:98. doi: 10.1186/s12957-024-03379-5 (PMC11020791; doi:10.1186/s12957-024-03379-5)
Supplement: Supplementary file 1 — Supplementary Material 1: Supplementary Table 1. Location of disease in staging imaging with positive findings. Description: where different types of disease spread was detected by different imaging modalities [file 12957_2024_3379_MOESM1_ESM.docx]

**Supplementary Table 1.** Location of disease in staging imaging with positive findings

|  | **Imaging Performed** | | | | |
| --- | --- | --- | --- | --- | --- |
| **Location of Disease** | **CT**  **N=13 (%)** | **MRI**  **N=8 (%)** | **SRS**  **N=3 (%)** | **MIBG N=0 (%)** | **^18^F-FDG PET/CT N=1 (%)** |
| **Distant Metastasis** |  |  |  |  |  |
| Liver | 7 (54) | 0 (0) | 0 (0) | 0 (0) | 1 (100) |
| Mesenteric Node | 0 (0) | 0 (0) | 1 (33) | 0 (0) | 0 (0) |
| **Local/Regional Disease** |  |  |  |  |  |
| Perirectal Lymph Node | 2 (15) | 5 (62) | 1 (33) | 0 (0) | 0 (0) |
| Primary Rectal Lesion | 5 (38) | 3 (38) | 1 (33) | 0 (0) | 0 (0) |

*CT* computed tomography; *MRI* magnetic resonance imaging; *SRS* somatostatin receptor scintigraphy; *MIBG* metaiodobenzylguanidine; ^18^F-FDG PET/CT fluorodeoxyglucose positron emission tomography
